# Electrochemical and Microbial Dissection of Electrified Biotrickling Filters

**DOI:** 10.3389/fmicb.2022.869474

**Published:** 2022-05-31

**Authors:** Benjamin Korth, Narcís Pous, Richard Hönig, Philip Haus, Felipe Borim Corrêa, Ulisses Nunes da Rocha, Sebastià Puig, Falk Harnisch

**Affiliations:** ^1^Department of Environmental Microbiology, Helmholtz Centre for Environmental Research, Leipzig, Germany; ^2^Laboratory of Chemical and Environmental Engineering (LEQUiA), Institute of the Environment, University of Girona, Girona, Spain

**Keywords:** microbial electrochemical technology, biologic nitrogen removal, denitrification, fixed bed reactor, cyclic voltammetry, metagenomic sequencing

## Abstract

Electrified biotrickling filters represent sustainable microbial electrochemical technology for treating organic carbon-deficient ammonium-contaminated waters. However, information on the microbiome of the conductive granule bed cathode remains inexistent. For uncovering this black box and for identifying key process parameters, minimally invasive sampling units were introduced, allowing for the extraction of granules from different reactor layers during reactor operation. Sampled granules were analyzed using cyclic voltammetry and molecular biological tools. Two main redox sites [−288 ± 18 mV and −206 ± 21 mV vs. standard hydrogen electrode (SHE)] related to bioelectrochemical denitrification were identified, exhibiting high activity in a broad pH range (pH 6–10). A genome-centric analysis revealed a complex nitrogen food web and the presence of typical denitrifiers like *Pseudomonas nitroreducens* and *Paracoccus versutus* with none of these species being identified as electroactive microorganism so far. These are the first results to provide insights into microbial structure-function relationships within electrified biotrickling filters and underline the robustness and application potential of bioelectrochemical denitrification for environmental remediation.

## Introduction

Electroactive microorganisms (EAMs) wire their metabolism to solid electron conductors or other cells beyond their cell membranes in a process called extracellular electron transfer (EET). For oxidative processes, EAM transfer electrons derived from substrate degradation to solid terminal electron acceptors, but EAM can also receive electrons from solid electron donors for performing reduction reactions (Logan et al., 2019). EAMs are harnessed in primary microbial electrochemical technologies (METs), with the technical devices being termed bioelectrochemical systems (BESs) (Schröder et al., 2015).

Bioelectrochemical systems are developed for numerous applications by offering electrodes as sinks (i.e., anode) or sources (i.e., cathode) of electrons for oxidation or reduction processes, respectively. Initially, they were investigated for coupling wastewater treatment and power production in microbial fuel cells (Pant et al., 2010). More recently, BES for microbial electroremediation went into focus as these represent a sustainable alternative to conventional remediation strategies (Pous et al., 2018). Instead of providing chemicals as electron donors and acceptors that are often limited in their availability, electrodes represent inexhaustible sources thereof. For instance, BESs were applied for remediating soil, sediment, surface water, and groundwater contaminated with nitrogen and sulfur compounds, heavy metals, aromatics, and chlorinated hydrocarbons (Sevda et al., 2018; Wang et al., 2020).

Nitrogen compounds, especially nitrate (${\text{NO}}_{3}^{-}$) and ammonium (${\text{NH}}_{4}^{+}$) are widespread pollutants in groundwater, mainly emanating from intensified agriculture and sewage disposal (Seitzinger and Phillips, 2017). Their removal using BES has been extensively studied (Rodríguez Arredondo et al., 2015), demonstrating the conduction of nitrification (Vilajeliu-Pons et al., 2018) and denitrification (Virdis et al., 2008) by EAM. Moreover, it was demonstrated that the anammox process (i.e., anaerobic ${\text{NH}}_{4}^{+}$ oxidation to N_2_) could be linked to electricity production (Shaw et al., 2020). Among the nitrogen pathways, bioelectrochemical denitrification is the most researched, and its suitability for treating, e.g., process water from the nutrition industry (Prokhorova et al., 2021), and also its robustness against other contaminants (Ceballos-Escalera et al., 2021) were demonstrated.

Bed electrodes, consisting of conductive graphite granules are easy-to-build and cost-effective electrodes, offering a high electrode surface area-to-reactor volume ratio that is essential for the performance enhancement of BES used for microbial electroremediation (Quejigo et al., 2019; Ceballos-Escalera et al., 2021). In general, fixed bed electrodes combine simple design, robustness, and straightforward operation (Quejigo et al., 2019), and several applications have been reported, including wastewater treatment with concomitant power production (Rabaey et al., 2005), degradation of azo dyes (Li et al., 2022), and nitrate removal (Pous et al., 2015). Furthermore, the combination of wetland technology with fixed bed electrodes is highly promising for decentralized wastewater treatment at the technical scale (Yadav et al., 2012). For instance, it was demonstrated that this technology can treat organic carbon and nitrogen contaminated water with high removal efficiencies (Srivastava et al., 2020).

**Figure 1:**
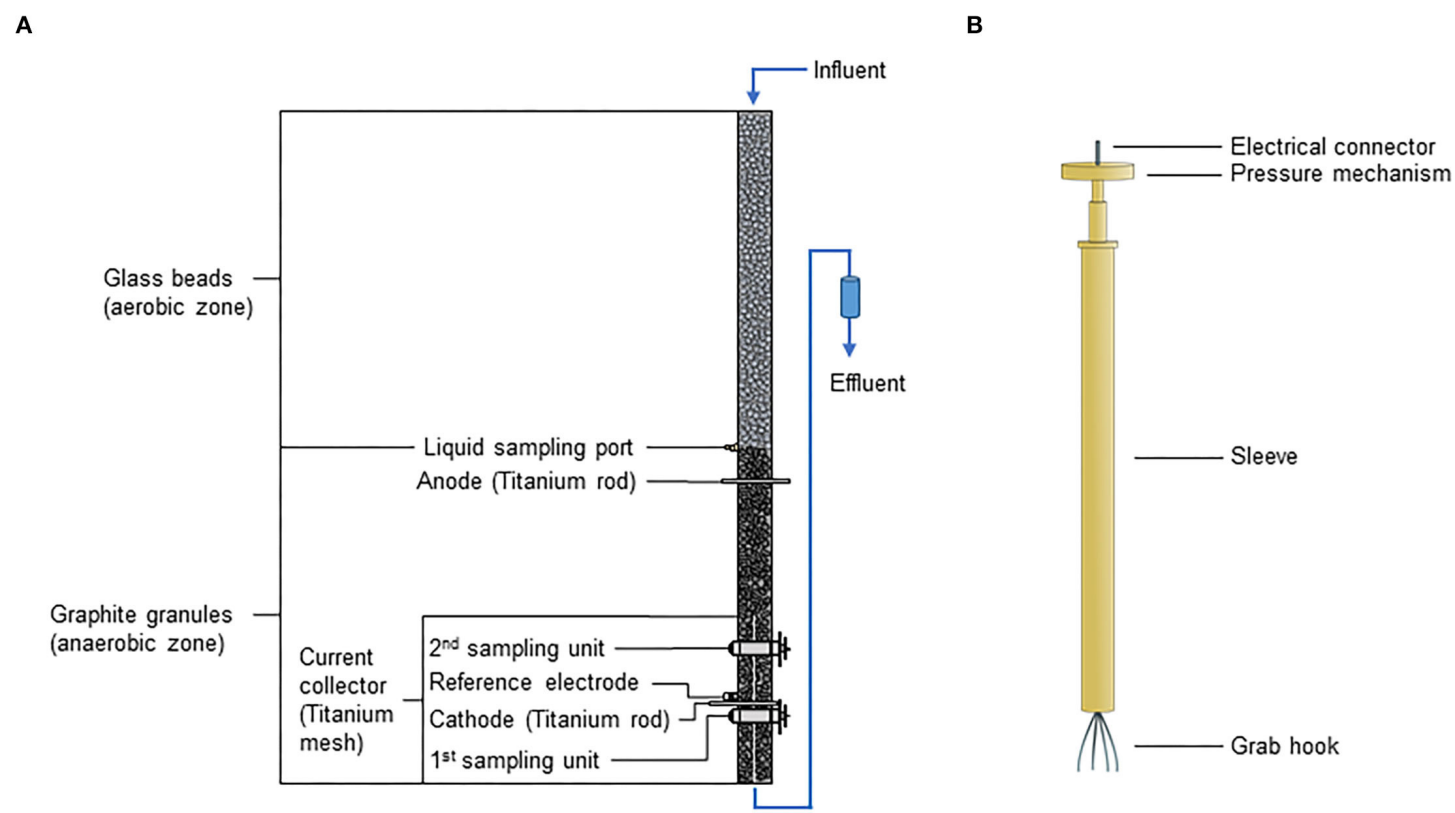
Schematic illustration of **(A)** electrified biotrickling filters with integrated sampling units and **(B)** e-clamp. The influent entered the reactor from the top leading to an aerobic zone in the reactor's upper half (filled with glass beads) enabling aerobic ammonia oxidation. As oxygen was consumed during ammonia oxidation, the lower half of the reactor (filled with graphite granules) became anaerobic allowing bioelectrochemical denitrification at the cathode. A titanium mesh connected to a titanium rod and a single titanium rod were used as cathodic and anodic current collectors, respectively.

In our previous study, electrified biotrickling filters with incorporated bed electrodes were proposed as cost-effective, easy-to-use, and robust BES for treating wastewater from aquaponics that usually contain a high ammonium content (Pous et al., 2021). Different reactor designs were tested based on ordinary biotrickling filters (i.e., plastic tubes filled with granular materials). The best performance was achieved with a design that combined an aerobic nitrification zone in the upper part with an electrified anaerobic zone at the bottom for denitrification. This reactor design hosted a titanium mesh as the current collector (CC) achieving ammonium (${\text{N-NH}}_{4}^{+}$) and total nitrogen (N-TN) removal rates of 94.0 ${\text{mgN-NH}}_{4}^{+}$L^−1^day^−1^ and 43.0 mgN-TN L^−1^ day^−1^, respectively. The study by Pous et al. represents a proof of principle that provided neither information on the microbial community nor the distribution of the microbial electroactivity within the electrified biotrickling filters. Therefore, electrified biotrickling filters were mimicked in this study to shed light on the microbiome of the electrified zone. To achieve this, in-house developed sampling units were integrated to enable a minimally invasive sampling of granules from the denitrification zone during operation (Figure 1A). Direct electrochemical measurements of sampled granules with the e-clamp (Quejigo et al., 2018), an analysis of the pH dependence of the bioelectrochemical denitrification activity, and functional potential analysis of the microbial community, and recovery of metagenome-assembled genomes were performed.

## Materials and Methods

### General Conditions

All chemicals were of analytical or biochemical grade. All provided potentials refer to the standard hydrogen electrode (SHE) by conversion from Ag/AgCl saturated KCl reference electrodes (+0.197 V vs. SHE). Experiments were performed at room temperature (23 ± 2°C).

### Assembly of Electrified Biotrickling Filters

Four reactors were constructed using customary polypropylene (PP) tubes of 1 m length and 4.6 cm inner diameter (Marley Deutschland GmbH, Germany) (Figure 1A). Three reactors (reactors 1–3) served as experimental replicates. One reactor was used as control, hosting no sampling units to test if the incorporation of these sampling units influences reactor performance, for instance, by the intrusion of oxygen in the anaerobic zone of the electrified biotrickling filters. Titanium rods (grade 2, 5 mm diameter, Goodfellow Cambridge Ltd., UK) connected to titanium meshes (25 × 5 cm, 0.28 mm wire, Alfa Aesar, U.S.A.) were used as CC for the working electrode (i.e., cathode). A titanium rod and an Ag/AgCl electrode (saturated KCl, +0.197 V vs. SHE, SE 11, Xylem Analytics Germany Sales GmbH & Co. KG Sensortechnik Meinsberg, Germany) were integrated as anodic CC and reference electrodes, respectively. The in-house developed granule sampling unit consisted of 2 cylinders made of polyvinyl chloride (PVC), the outer cylinder of the sampling unit was made of acrylic glass and fixed with glue to the PP tube. The sampling window was 12 × 8 mm, and the sampling pocket of the inner cylinder had a depth of 5 mm (Quejigo et al., 2021). At the bottom of the reactor, a PVC cap with a tube connector as an effluent port and a titanium mesh (5 × 5 cm) were integrated to prevent clogging of this port by granules. All components were glued to the PP tube using vulcanizing glue (Tangit PVC-U Plus, Henkel AG & Co. KgaA, Germany).

Graphite granules (model 00514, Enviro-cell, Germany) were sieved to select granule sizes of 1.5–5.0 mm. Granules were consecutively washed with tap water, 1 M NaOH (for 24 h), tap water, 1 M HCl (for 24 h), and distilled water. The lower and the upper half of the reactors were filled with granules (703 ± 29 g in 50 cm resulting in a liquid net volume of 285 ± 5 ml) and glass beads (diameter 2 mm, 1,420 ± 42 g in 50 cm, resulting in a liquid net volume of 331 ± 24 ml), respectively. The simple and cost-effective open reactor design resulted in an aerobic zone in the upper half of the reactor enabling ammonia oxidation. Consequently, oxygen was consumed along the reactor so that the lower half was filled with conductive granules, which became anaerobic allowing bioelectrochemical denitrification therein. The control reactor was filled with 789 g granules (liquid net volume of 327 ml) and 1,383 g glass beads (liquid net volume of 365 ml) in the lower and upper half, respectively.

The medium was stored in a 20 L polyethylene canister and pumped with a four-channel pump (REGLO *Analog*, ISMATEC, Germany) using pumping tubes (inner diameter = 1.52 mm, Tygon LFL, SC0424, ISMATEC, Germany) and tubes (inner diameter = 1.6 mm; TYGON® E-3603, Saint-Gobain Performance Plastics, France). The tubes were sterilized by pumping 2.5% H_2_SO_4_ for 12 h, followed by cleaning with distilled water before use. The canisters were washed with 2.5% H_2_SO_4_ and distilled water before use.

### Reactor Start-Up

In total, 2 L effluent from previously described biotrickling filters (Pous et al., 2021) were centrifuged (10 min, 10,000 g). Cell pellets were resuspended in 1.5 L synthetic aquaculture effluent, containing 50 mg L^−1^ ${\text{N-NH}}_{4}^{+}$ and 50 mg L^−1^ ${\text{N-NO}}_{3}^{-}$, which served as the medium for all experiments (0.162 g Na_2_HPO_4_, 1,072 g KH_2_PO_4_, 0.1 g MgSO_4_ × 7 H_2_O, 0.015 g CaCl_2_, 0.25 g NaCl, 1.05 g NaHCO_3_, 0.19 g NH_4_Cl, 0.30 g NaNO_3_, 12.5 ml vitamin solution, and 12.5 ml trace element solution dissolved in 975 ml distilled water) (Pous et al., 2021). In total, 350 ml of this inoculum solution were added to every reactor. The cathode potential (*E*_Cat_) was controlled with a potentiostat (VSP, BioLogic Sciences Instruments, France) and initially adjusted to −100 mV vs. SHE. After 2 days without pumping, reactor liquid was pumped in a closed-loop mode with a hydraulic retention time (HRT) of 24 h. The inflow and outflow were at the top and bottom of reactors, respectively. On day 14, 350 ml of medium with 100 mg L^−1^ of ${\text{N-NH}}_{4}^{+}$ and 100 mg L^−1^ of ${\text{N-NO}}_{3}^{-}$ was added, and the HRT was adjusted to 3 h for 2 more weeks for increasing mixing and facilitating biomass growth.

### Continuous Reactor Operation

On day 30, the continuous operation was initiated with 12 h HRT and the synthetic aquaculture effluent. As bioelectrochemical denitrification requires anoxic conditions, 20 L of fresh medium were sparged with N_2_/CO_2_ (80%:20%) for 2 h. On day 54, the liquid level was adjusted to 75% of reactor height, and HRT was set to 24 h approximating operational parameters applied in the previous study (Pous et al., 2021). Due to the limitations of the potentiostat (refer to the Section Reactor Operation and Removal Performance), the initial *E*_Cat_ of −100 mV could not be applied during continuous mode and had to be gradually adapted. After 100 days, this adaptation phase was finished, and the operational phase started with an applied *E*_Cat_ of +250 mV. The pH of the influent during the operational phase was 7.0 ± 0.2.

Granules were extracted from day 129 onward for performing electrochemical and molecular biological analyses. Electrochemical data from electrified biotrickling filters were collected every 10 min using EC-Lab V11.31 (BioLogic Sciences Instruments, France).

### Chemical Analyses

Ammonium (${\text{N-NH}}_{4}^{+}$) was photometrically measured with an alkaline dichloroisocyanurate-salicylate-nitroprusside-based assay at a wavelength of 660 nm (DIN ISO 105661999-4). Nitrite (${\text{N-NO}}_{2}^{-}$) was photometrically measured with sulfanilamide and N-(1-naphthyl)ethylenediamine at acidic conditions at a wavelength of 540 nm (DIN EN 26777). Nitrate (${\text{N-NO}}_{3}^{-}$) was determined by means of total oxidized nitrogen (TON) (EN ISO 13395). After reducing nitrate to nitrite with hydrazine under alkaline conditions, the absorbance at 540 nm was measured and related to TON. Subsequently, the nitrate concentration was calculated by subtracting nitrite concentration from TON. Ammonia, nitrate, and nitrite were determined with Gallery Plus Discrete (Thermo Fisher Scientific Inc., U.S.A.) using calibration curves.

Ammonium removal and total nitrogen (N-TN) removal were calculated using the concentration differences between influent and effluent of ammonium and all determined nitrogen species (i.e., ${\text{N-NH}}_{4}^{+}$, ${\text{N-NO}}_{3}^{-}$, and ${\text{N-NO}}_{2}^{-}$), respectively. The ammonium removal rate (${\text{N-NH}}_{4\text{RR}}^{+}$) and total nitrogen removal rate (N-TN_RR_) were calculated considering the total net volume of reactors and flow rate.

The dissolved oxygen concentration in the effluent was measured using a WTW FDO® 925 sensor (Xylem Analytics Germany Sales GmbH & Co. KG. Germany).

### e-Clamp

The e-clamp was an advancement from the originally reported version (Quejigo et al., 2018). The outer e-clamp parts were made of polyether ether ketone (PEEK) (Figure 1B). The grab hook consisted of four spring steel wires (V4A 1.4571, diameter 0.6 mm, Febrotec GmbH-Federn, Germany), which were pressed together in a stainless steel sleeve that also represented the electrical connector at the top of the e-clamp. The clamp mechanism was achieved by integrating two springs (stainless steel 1.430, wire diameter 0.45 mm, spring diameter 4.05 mm, and spring length 17.7 mm, Federntechnik Knörzer GmbH, Germany). The electrically conductive parts inside the PEEK sleeve and the grab hook were insulated with acrylic varnish. Only 1 cm of each grab hook wire was electrically conductive.

### Cyclic Voltammetry

Granules were extracted for performing cyclic voltammetry using the sampling units, fixed with the grab hook of the e-clamp, and directly transferred to four-neck round-bottom flasks with an integrated three-electrode setup (Supplementary Figure S1) (Quejigo et al., 2021). The e-clamp was pierced through a silicone stopper and represented the working electrode, 250 ml medium was purged for 25 min with N_2_/CO_2_ (80%:20%) before the e-clamp was inserted. The headspace was constantly purged, the medium (synthetic aquaculture effluent containing 50 mg L^−1^ ${\text{N-NH}}_{4}^{+}$ and 50 mg L^−1^ ${\text{N-NO}}_{3}^{-}$) was stirred with a magnetic stirrer, and a temperature of 35°C was maintained during CV. 20 ml of the same medium but without ${\text{NH}}_{4}^{+}$ and ${\text{NO}}_{3}^{-}$ was used in the counter electrode chamber. CV was conducted from −600 to +600 mV with a scan rate of 1 mVs^−1^ using a potentiostat (MPG-2, BioLogic Sciences Instruments, France*)*. Between CVs, the pH of the media in the main chamber was adjusted using 2 M HCl, 4 M KOH, and a pH meter (LAQUAtwin pH-22, HORIBA Advanced Techno, Japan). After experiments, granules were stored at −20°C. To determine the granule weight and thus gravimetric current density, all granules were dried at 80°C overnight after DNA extraction (Section Molecular Biological Analyses) and stored in a desiccator until weight stabilization. Electrochemical data were collected with EC-Lab V11.31 and analyzed with OriginPro 2019 (Version 9.6.0.172, 64-bit).

During the analysis of the pH influence on the normalized gravimetric current density (refer to the Section Analyzing the Influence of pH on Bioelectrochemical Denitrification), the Grubbs test was used to remove outliers at a significance level of 0.01 using OriginPro 2019.

### Molecular Biological Analyses

Genomic DNA was extracted from sampled granules with the NuceloSpin Tissue Kit (Macherey-Nagel GmbH & Co. KG, Germany) for terminal restriction fragment length polymorphism (TRFLP) analysis as previously described (Korth et al., 2020) and metagenomics sequencing. By doing so, the amounts of lysis and extraction buffer were adjusted to cover all granules within a reaction tube. The amount of ethanol for DNA precipitation was adjusted accordingly. To perform genome-centric analysis of the functional potential present in the microbial community, two granule samples were submitted to GENEWIZ Germany GmbH (reactor 1, 1st sampling unit, day 253 and reactor 3, 1st sampling unit, day 283) for metagenome sequencing. The metagenomics libraries were prepared using NEBNext® Ultra (New England BioLabs Inc., U.S.A.) according to the manufacturer's instructions. After these metagenomics libraries were sequenced with an Illumina NextSeq 500 (Illumina Inc., U.S.A.) according to the manufacturer's instructions, ~25 million paired-end reads (150 bp each) were obtained prior to quality control. To recover metagenome-assembled genomes (MAGs), MetaWRAP version 1.1.7 (Uritskiy et al., 2018) was used according to the developer's instructions. Then, we selected only MAGs with quality scores higher than 50 (quality score = completeness – 5 × contamination) (Parks et al., 2017). Taxonomy was assigned to the MAG using GTDB-Tk v0.3.3 (Chaumeil et al., 2020). To define the operational taxonomic units (OTUs), the MAGs were grouped per unique taxonomy and clustered using hierarchical agglomerative clustering with the average nucleotide identity (ANI) distances. Generally, 0.99 ANI distance was used as the threshold as a proxy of the species level.

To study the functional capacity for the nitrogen cycle (KEGG modules: M00531, M00530, M00529, M00528, and M00804) (Kanehisa et al., 2016), the recovered genomes were annotated using Prokka version 1.14.5 (Seemann, 2014). To expand the default Prokka database, a custom database was created by downloading all the proteins related to KEGG modules (download in June 2021). After annotation, all predicted protein names were manually standardized, and a table of presence or absence was created for each OTU.

## Results and Discussion

### Reactor Operation and Removal Performance

Three electrified biotrickling filters with integrated sampling units and one control reactor without were operated for around 300 days. The first 100 days were the inoculation and adaption phase in which the flow rate, liquid level, and cathode potential (*E*_Cat_) were adjusted for balancing process parameters. The application-oriented, simple, and cost-effective open reactor design resulted in an aerobic zone filled with glass beads in the upper reactor part (Figure 1A). In this study, aerobic ammonium oxidation occurred according to the previous publication (Pous et al., 2021). However, if oxygen diffuses to the lower electrified reactor zone filled with graphite granules, it is electrochemically reduced. This leads to high negative currents that are unrelated to the desired process of bioelectrochemical denitrification. Therefore, the initially applied *E*_Cat_ of −100 mV, known to facilitate bioelectrochemical nitrate reduction (Pous et al., 2014), was stepwise changed during the adaptation phase to +250 mV. After 100 days, the operational phase started, and the data obtained therefrom were used for all analysis. Despite the more positive *E*_Cat_, removal rates of ammonium (${\text{N-NH}}_{4\text{RR}}^{+}$) and total nitrogen (N-TN_RR_) of 34.0 ± 12.1 gN m^−3^ day^−1^ and 43.4 ± 28.4 gN m^−3^ day^−1^, respectively, were achieved (Table 1). An activity stratification within electrified biotrickling filters was in place by comparing process parameters derived from sampling influent, middle liquid port, and effluent. Specifically, although the main share of ammonium was oxidized in the upper aerobic zone, nitrate was mainly reduced in the electrified anaerobic zone at the bottom. Furthermore, considerable amounts of nitrite (30–40 ${\text{mgN-NO}}_{2}^{-}$ L^−1^) were detected in the middle at the beginning of the operational phase (~days 100–125). In the following days, only minor amounts of nitrite were detected in the reactor middle (1.9 ± 3.7 ${\text{mgN-NO}}_{2}^{-}$ L^−1^ for days 130–300) and the effluent (2.4 ± 3.9 ${\text{mgN-NO}}_{2}^{-}$ L^−1^ for days 130–300) indicating an improved total nitrogen removal due to growth of denitrifying microorganisms (see Supplementary Figures S2–S5 for the evolution of process parameters of all reactors).

**Table 1:** Comparison of main process parameters obtained in this study and Pous et al. (2021).

	**Electrified biotrickling filter (*n* = 3)**	**Control reactor (*n* = 1)**	**Pous et al., 2021 (*n* = 1)**
Cathode potential, *E*_Cat_(mV)	243.2 ± 68.0	251.2 ± 25.3	0 ± 100
Ammonium removal rate, ${\text{N-NH}}_{4\text{RR}}^{+}$(gN m^−3^ day^−1^)	34.0 ± 12.1	34.0 ± 10.9	38.0 ± 2.0^a^
Total nitrogen removal rate, N-TN_RR_(gN m^−3^ day^−1^)	43.4 ± 28.4	43.1 ± 25.9	31.8 ± 6.2^a^
Effluent pH	7.2 ± 0.4	7.3 ± 0.3	7.4 ± 0.2

a *Obtained with hydraulic retention time (HRT) of 1.2 days and 75% liquid level representing not the best reactor performance achieved in this study*.

Similar removal rates were observed for the control reactor (Table 1), showing a minor influence of the sampling units on the overall reactor performance. Moreover, the observed ${\text{N-NH}}_{4^{+}\text{RR}}$ was similar and N-TN_RR_ even higher as reported by Pous et al. using the similar HRT and liquid level (Pous et al., 2021) (Table 1). Yet, it is noted that in addition to the cathode potential, the medium composition of both studies was different. The medium used in this study contained ammonium and nitrate. Typically, aquaculture effluents contain mainly ammonium but almost no nitrate (Yin et al., 2018). We amended the medium in this study by nitrate to foster an electrified zone that performs bioelectrochemical denitrification and to ensure a subsequent electrochemical analysis. Anaerobic conditions within reactors were verified by measuring the dissolved oxygen (DO) content in the effluent. It amounted to 0.23 ± 0.07 mg L^−1^ (4 measurements per reactor between days 198 and 256) and thus was comparable to the proof of concept (Pous et al., 2021).

### Cyclic Voltammetry With Sampled Graphite Granules

During the operational phase starting at day 129, graphite granules were collected from the electrified biotrickling filters *via* the minimally invasive sampling units. Subsequently, cyclic voltammetry was performed with sampled granules using the e-clamp in the presence of ammonium and nitrate (Supplementary Figure S1). In total, 93 granule samples were taken from the reactors during the operational phase. In addition, when terminating the reactor operation, all reactors were sampled along the vertical axes of the graphite granule bed (i.e., 6 sample spots regularly distributed along 50 cm of graphite granule bed, Supplementary Figure S6A), yielding further 24 granule samples. However, graphite granules are porous and only exhibit limited physical contact, resulting in an inhomogeneous redox potential (Newman and Tobias, 1962). Thus, the electrochemical driving force for EET and the ecological niche for EAM is not identical at all granules. This leads to different biomass growth at granules, and thus only ~60% of the sampled granule exhibited an electrochemical signal that could be analyzed.

#### Identifying Two Main Redox Sites for Bioelectrochemical Denitrification

**Figure 2:**
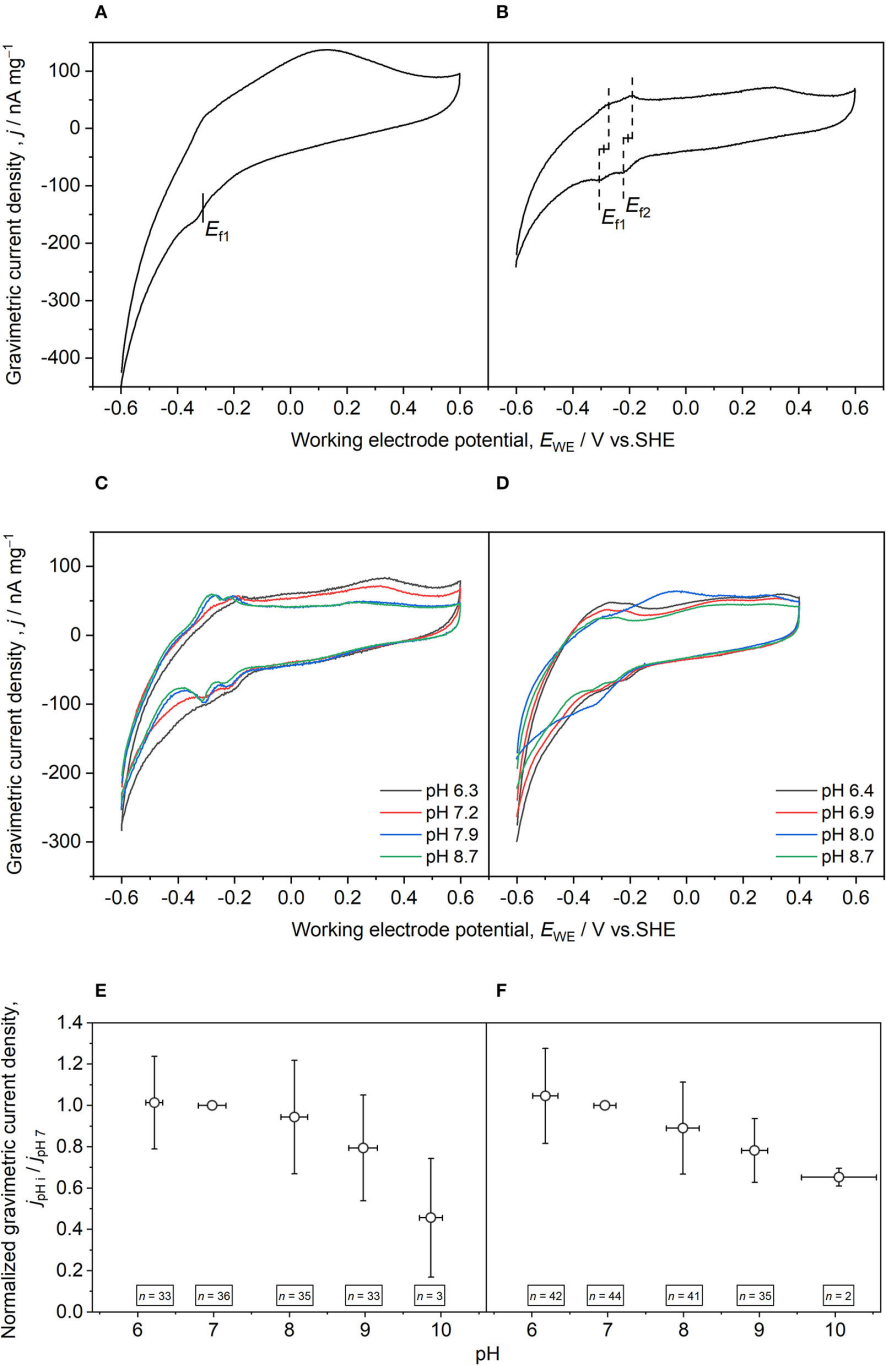
Exemplary cyclic voltammograms recorded with granules sampled during the operational phase for determining formal potentials **(A,B)** and the bioelectrochemical response to different pH values **(C–F)**. **(A)** Reactor 1, 2nd sampling unit [more distant to current collector (CC)], day 247. The formal potential of the first redox site (*E*_f1_) was determined by analyzing the respective inflection point. **(B)** Reactor 2, 1st sampling unit (close to CC), day 253. Formal potentials of the first and second redox sites (*E*_f2_) were determined by calculating the arithmetic mean of the anodic and cathodic peak potentials. **(C)** Reactor 2, 1st sampling unit (close to CC), day 253. **(D)** Reactor 3, 2nd sampling unit (more distant to CC), day 310. For experiments at different pH values (i), gravimetric current density (*j*) at formal potentials **(E)** *E*_f1_ and **(F)** *E*_f2_ were quantified at every pH (*j*_pHi_) and normalized with the gravimetric current density at pH 7 (*j*_pH7_). Per definition, the normalized gravimetric current density was 1 at pH 7. The scan rate during CV was 1 mV s^−1^, only 3rd scans are shown. Errors bars indicate standard deviations. *n* indicates the number of analyzed granules.

Two main redox sites were identified with formal potentials of −288 ± 18 mV (*E*_f1_, *n* = 25) and −206 ± 21 mV (*E*_f2_, *n* = 27) (Table 2; Figures 2A,B). These *E*_f_ are in the range of already observed values for denitrifying microbial cathodes (Yu et al., 2015; Vilar-Sanz et al., 2018; Wang and Zhang, 2019). *E*_f2_ determined from the 2nd sampling unit was significantly more negative than observed for the 1st sampling unit being closer to the current collector (*p* = 0.007). No significant difference among the sampling units was observed for *E*_f1_ (*p* = 0.069). This finding indicates that the titanium mesh that was integrated into the reactors for a more homogeneous redox potential distribution within the granule bed leads to comparable ecological niches and hence similar *E*_f1_. The similar gravimetric current densities ($j_{\text{Ef}\frac{1}{\text{Ef}}2}$, current normalized to the weight of dried granules) achieved during CV for both sampling units support this (Table 2). Nevertheless, the titanium mesh covered only two dimensions (Figure 1A). This was not sufficient for a complete even polarization of the granule bed, as may be deduced from the shift of *E*_f2_. We speculated that this was related to EET for improved utilization of the available redox conditions. The redox potential within the granule bed becomes more positive with more distance to the cathodic CC. Thus, a shift of the formal potential of the electron-accepting redox site to more negative values would result in a stronger redox gradient within the electron transport chain of denitrifiers and hence a higher catabolic energy harvest (Kracke et al., 2015). This adaptation would occur on the detrimental effect that the EET rate and maybe also the rate of electron conduction in the bed are decreased. Furthermore, *E*_f_ could also be influenced by different oxygen concentrations. As the second sampling unit was closer to the aerobic zone, more oxygen was likely present compared with the 1st sampling unit.

**Table 2:** Overview about formal potentials (*E*_f_) and corresponding gravimetric current densities (*j*) obtained from granules sampled from the electrified biotrickling filters *via* sampling units.

	**1st sampling unit (close to CC)**		**2nd sampling unit**	
*E*_f1_(mV)	−281 ± 14	(*n* = 13)	−294 ± 19	(*n* = 12)
*j*_Ef1_(nA mg^−1^)	−151 ± 98		−155 ± 96	
*E*_f2_(mV)	−199 ± 18	(*n* = 18)	−221 ± 17	(*n* = 9)
*j*_Ef2_(nA mg^−1^)	−137 ± 108		−133 ± 93	
pH	7.0 ± 0.3	(*n* = 23)	7.0 ± 0.3	(*n* = 17)

*j was determined at the respective E_f_. n indicates the number of sampled granules*.

#### Consequences of Sampling Granules and e-Clamp Analysis

Determination of *E*_f1_ and *E*_f2_ was based on an analysis of inflections points (Figure 2A) and pairs of redox peaks (Figure 2B) that occurred equally distributed in CV experiments. The occurrence of paired redox peaks indicates non-turnover conditions (i.e., absence of the substrate), although the assumed substrate nitrate is readily available. We hypothesized mainly three reasons for the occurrence of turnover and non-turnover redox signals in the presence of nitrate, namely, (1) The redox sites are not exclusively associated with nitrate reduction but also with the reduction of denitrification intermediates (e.g., nitrite and nitrous oxide). These are unavailable from the beginning of the CV experiments but may become available to small extents during CV. Although the observed formal potentials were too positive for being related to nitrite reduction, nitrous oxide reduction seems reasonable (Vilar-Sanz et al., 2018). (2) Although the granule sampling was designed to be minimally invasive, it cannot be prevented that the biofilm integrity is disturbed during sampling so that cells may have lost contact with other cells or the granules. Consequently, some redox sites are not wired to the metabolism by actively participating in EET and show oxidation and reduction (Peng et al., 2016; Quejigo et al., 2021). Therefore, the ratio between rates of EET, intracellular electron transfer, metabolism, and especially their joints were changed, leading to apparent non-turnover signals. (3) Single granules were subjected to different mass transfer regimes when comparing CV experiments with e-clamp (i.e., radial diffusion) and their embedding within the bed electrode (i.e., mainly planar diffusion). Thus, potential limitations due to, for instance, substrate supply and counter ion transport were decreased, leading to different kinetics (Quejigo et al., 2021).

#### Vertical Analysis of the Bed Electrodes at the End of the Operational Phase

At the end of the operational phase, granules were extracted along the vertical axes of the bed electrode of the three electrified biotrickling filters with and the control reactor without integrated sampling units at 6 equally distributed positions. Although the sample size of this experiment is relatively small, the results suggest that there are no substantial differences between the reactors with sampling units and the control reactor. The distribution, frequency, and redox potentials of cathodic and anodic redox sites are comparable (Supplementary Figures S6B–D). Furthermore, the occurrence of redox sites *E*_f1_ and *E*_f2_ at granules closer to the anodic CC indicates that also zones being more distant to the cathodic CC (titanium rod and mesh) contributed to bioelectrochemical denitrification. Although the current contributions of e-clamp (Supplementary Figure S7A) and non-inoculated granules can be distinguished (Supplementary Figure S7B), a differentiation between faradaic and capacitive currents at sampled granules is challenging. We assigned this to the infinite capacitance of the blank carbon and the finite capacitance when it is at least partly covered with biofilm (Kretzschmar and Harnisch, 2021). Consequently, a precise vertical mapping of the denitrification activity and contribution to N-TN_RR_ in the granule bed can hardly be achieved. Nevertheless, considering the prevailing autotrophic growth conditions in the electrified biotrickling filters and the resulting limited biomass growth, the observed *j* seems reasonable when being compared with similar e-clamp experiments with granules from *Geobacter* enrichment biofilms cultivated in acetate-fed fixed bed electrodes [~2 μA mg^−1^ during CV recorded with 1 mV s^−1^ (Quejigo et al., 2021)].

#### Indications for Electrified Anammox

For ~10% of the sampled granules during the operational phase, oxidation peaks in the potential range from 0 to +350 mV were observed (Supplementary Figures S7C–E). Oxidation peaks at a similar range were described for EET-dependent anammox (Shaw et al., 2020) (also refer to the Section Molecular Biological Analysis of Sampled Granules) but not for bioelectrochemical ammonium oxidation (Vilajeliu-Pons et al., 2018). Although the results indicate that the electrode bed in the anaerobic upper zone of the reactor possessed ammonia oxidation capabilities, probably *via* EET-dependent anammox, their rare occurrence in e-clamp experiments prevented a systematic study of this phenomenon.

### Analyzing the Influence of pH on Bioelectrochemical Denitrification

During the electrochemical analysis of sampled granules (refer to the Section Cyclic Voltammetry With Sampled Graphite Granules), pH was sequentially changed in the range of 6–10, while CV was performed for each pH (Figures 2C,D). For analyzing pH dependence, *j* at the beforehand determined formal potentials *E*_f1_ and *E*_f2_ were quantified at every pH (*j*_pHi_) and normalized to the gravimetric current density at pH 7 (*j*_pH7_). Intriguingly, both redox sites exhibit a nearly constant current consumption in the pH range 6–8 (Figures 2E,F), representing the typical pH optimum for microbial denitrification (Tiedje, 1988). Only at pH 9, the normalized *j* decreased below 80% compared with pH 7, which was continued to pH 10 achieving around 46% (*E*_f1_) and 65% (*E*_f2_) activity. Therefore, electrified biotrickling filter seems to be an appropriate treatment method for aquaponics wastewater as pH 6–7 is the optimal operating range of aquaponic plants. In addition, it also provides a safety margin in case of unwanted alkalinization (Food Agriculture Organization of the United Nations, 2014). Although certain pH robustness is known for denitrifying microbiomes reporting microbial denitrification at up to pH 11–12 (Albina et al., 2021), this is, to our knowledge, not reported for bioelectrochemical denitrification. This broad pH range further supports the biotechnological potential of bioelectrochemical denitrification and extends its fields of application to moderate alkaline environments. As CV results indicate that the pH optimum is in the pH range 6–8, electrified biotrickling filters are suited for application in aquaponics systems. Plants prefer slightly acidic conditions (pH 6.0–6.5) improving nutrient availability and most fishes exhibit a pH tolerance range of 6.0–8.5 (Food Agriculture Organization of the United Nations, 2014).

In most cases, *E*_f1_ and *E*_f2_ shifted to more negative values for more alkaline pH during CV experiments (Figure 2C) following a typical Nernst behavior and the redox-Bohr effect already described for anodic EAM and their cytochromes (Morgado et al., 2012). To assess the effect of formal potential shift on the pH influence on bioelectrochemical denitrification, normalization of *j* was also performed by quantifying *j* at the shifted formal potentials for all studied pH values. This analysis revealed an even broader pH optimum. For *E*_f1_, *j* was comparable for pH 6–9 and decreased to 56% at pH 10, but in the case of *E*_f2_, no apparent differences could be observed for the tested range of pH 6–10 (Supplementary Figure S8). However, as the formal potentials shifted to more negative values, it cannot be excluded that the hydrogen evolution reaction contributed to the current. Furthermore, it can only be speculated if the applied pH range already led to conformational changes of the redox-active proteins and other cellular components affecting cathodic EET.

### Molecular Biological Analysis of Sampled Granules

Sampled granules were also analyzed with molecular biological methods for investigating the microbial community composition within the granule bed of the electrified biotrickling filters. Due to the reactors' autotrophic conditions and the resulting limited growth, some granule samples were pooled for obtaining sufficient DNA required for molecular analysis (1–3 granule samples were used for DNA extraction).

#### Terminal Restriction Fragment Length Polymorphism Analysis

First, the TRFLP analysis was conducted to overview the microbial community dynamics during the operational phase. TRFLP results indicate a limited microbial diversity and variability during the operational phase. Only a few terminal restriction fragments (TRFs) appear with comparable relative abundances during the operational phase in all reactor replicates indicating the existence of a microbial core community for bioelectrochemical denitrification (Supplementary Figure S9).

Interestingly, similar TRF patterns appear in all positions of the electrified biotrickling filters during the vertical analysis at the end of the operational phase (Supplementary Figures S6A, S10). This indicates that the incorporated CC (metal mesh covering *ca*. 50% of the vertical axis of the granule bed) led to a homogeneous potential distribution resulting in a comparable distribution of the microbial community. It is of note that the vertical dissection of the control reactor yielded a comparable distribution dominated by few TRF suggesting a similar microbial core community. This provides further proof of the negligible impact of the integrated sampling units on the microbiome (Supplementary Figure S10D).

However, in addition to the redox potential gradient, other gradients (e.g., ammonia, nitrate, intermediates of nitrogen pathways, and oxygen) exist within the granule bed that influence microbial growth and potentially covered a possible microbial stratification induced by the redox potential.

#### Metagenomic Sequencing and Analysis

**Figure 3:**
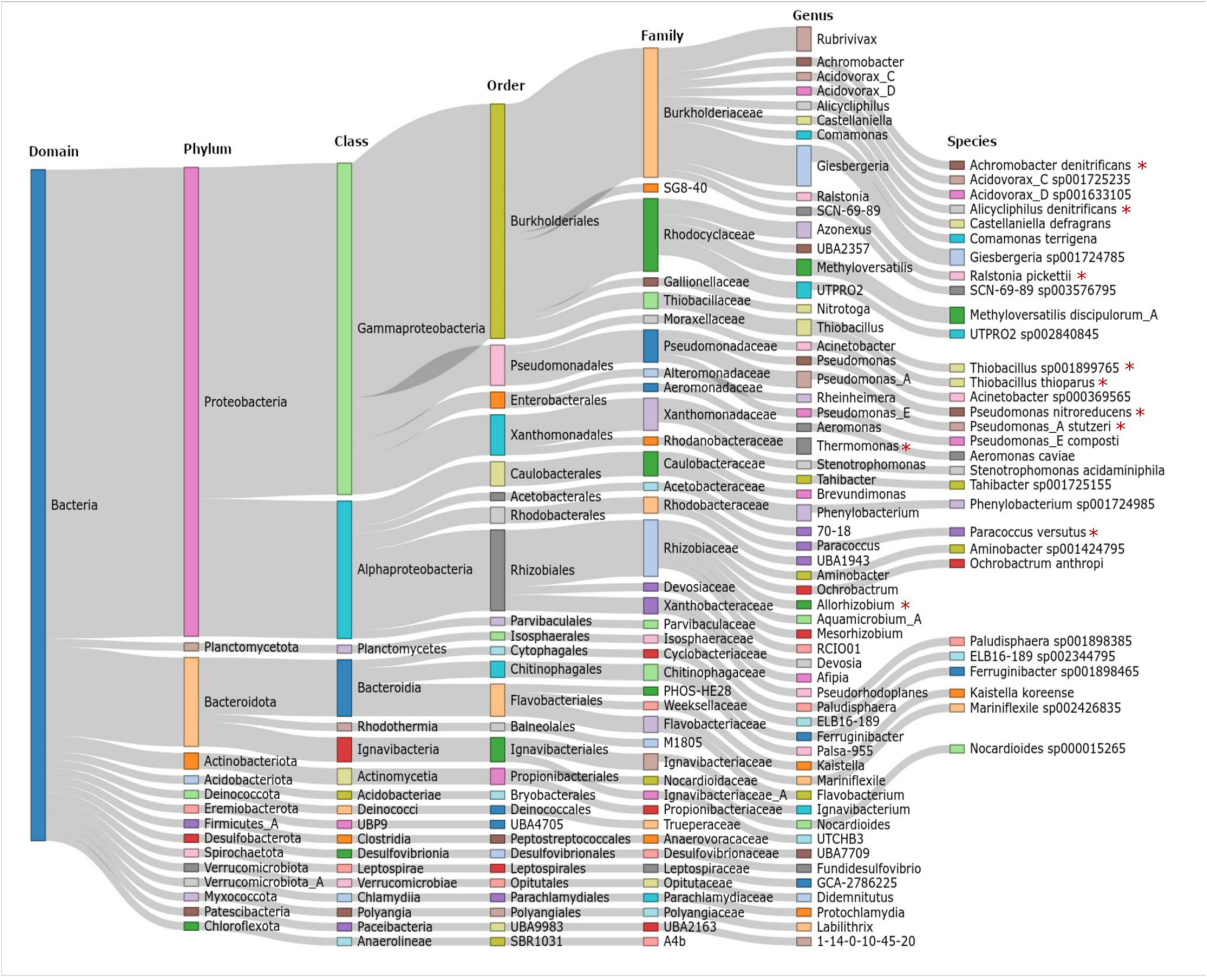
Sankey plot showing the taxonomic diversity of the 83 operational taxonomic units (OTUs, proxy for species level) recovered from the metagenomes. Red asterisks indicate species and genera that are described to perform denitrification reactions.

Subsequently, a genome-centric analysis of the functional potential of the microbial community was performed by analyzing two representative granule samples (Section Molecular Biological Analyses, a taxonomy table is provided in Supplementary Material 2). The results demonstrate the complexity of the microbial community within the granule bed of electrified biotrickling filters, as 83 OTUs (proxy for the species level) were assigned (Figure 3). Among the identified species, several were described to perform nitrate reduction or reduction of denitrification intermediates, for instance, *Alicycliphilus denitrificans* (Mechichi et al., 2003), *Ralstonia pickettii* (Ryan et al., 2007), and *Paracoccus versutus* (Zhang et al., 2018). However, to the best of our knowledge, none of these species has been assigned to perform cathodic EET so far. Instead, only soluble electron donors were described like cycloalkanes (Mechichi et al., 2003) and aromatics (Ryan et al., 2007) representing unlikely substrates (e.g., produced by syntrophic interactions) in the electrified biotrickling filters. Interestingly, the genus *Thiobacillus* is described to host electroactive members (Pous et al., 2014). However, the *Thiobacillus thioparus* identified in this study is not identified as an EAM so far. In some cases, the genome-centric analysis could not be conducted beyond the genus level but, in this case, potential denitrifiers (*Thermomonas* and *Allorhizobium*) (Zhao et al., 2017; Safonov et al., 2018) and nitrifiers (*Nitrotoga*) (Kitzinger et al., 2018) were also identified.

The recovered genomes were annotated, and proteins were identified to study the functional capacity for performing reactions in the nitrogen cycle (Supplementary Material 3). By doing so, all enzymes of the dissimilatory nitrate reduction and the denitrification pathway could be identified. Furthermore, few enzymes of the assimilatory nitrate reduction, nitrification, complete nitrification (commamox), and anammox pathways were identified illustrating the complexity of the nitrogen food web within the electrified biotrickling filters.

## Conclusion

The granule bed of electrified biotrickling filters was investigated by applying electrochemical and molecular biological methods. Minimally invasive sampling allowed dissecting of the bed cathode by analysis of single granules during reactor operation. Cyclic voltammetry revealed two main redox sites and showed that the bioelectrochemical denitrification activity is high in a broad pH range (i.e., 6–10). This robustness is advantageous for integrating electrified biotrickling filters into aquaponics and underlines the application potential of bioelectrochemical denitrification. Furthermore, cyclic voltammetry results showed that different nitrogen pathways vertically merge, resulting in reaction gradients within the electrified biotrickling filter. In connection with the identification of different nitrogen pathways, these observations suggested functional redundancies within the system being beneficial for the application (Koch et al., 2017). Molecular biological analyses of sampled granules also indicated the existence of a core community and presence of typical denitrifiers (e.g., *A. denitrificans, R. pickettii*, and *P. versutus*), but none of these species was identified as an EAM so far. Although this study provides the first insights into the inner workings of electrified biotrickling filters, further experiments are required to obtain more detailed information, especially about the microbial composition, allowing targeted steering of reactors for better performance.

## Data Availability Statement

The datasets presented in this study can be found in online repositories. The metagenome sequence reads of this study were deposited in the European Nucleotide Archive (ENA) database under accession no. PRJEB48350.

## Author Contributions

BK: conceptualization, investigation, formal analysis, methodology, and writing—original draft. NP: conceptualization, methodology, and writing—review and editing. RH and PH: data curation, investigation, and methodology. FC: formal analysis and writing—review and editing. UN: methodology, supervision of metagenomic analysis, and writing—review and editing. SP: conceptualization, funding acquisition, supervision, project administration, and writing—review and editing. FH: methodology, funding acquisition, supervision, project administration, and writing—review and editing. All authors contributed to the article and approved the submitted version.

## Funding

This research was carried out in the project Wireless Aquaponic Farming in Remote Areas: A Smart Adaptive Socio-Economic Solution (WAFRA) funded within the 7th Framework Program (ERANETMED). This study was supported by the Helmholtz Association in the frame of the Integration Platform Tapping Nature's Potential for Sustainable Production and a Healthy Environment at the UFZ. SP is a Serra Húnter Fellow (UdG-AG-575) and acknowledges funding from the ICREA Academia Award.

## Conflict of Interest

The authors declare that the research was conducted in the absence of any commercial or financial relationships that could be construed as a potential conflict of interest.

## Publisher's Note

All claims expressed in this article are solely those of the authors and do not necessarily represent those of their affiliated organizations, or those of the publisher, the editors and the reviewers. Any product that may be evaluated in this article, or claim that may be made by its manufacturer, is not guaranteed or endorsed by the publisher.

## References

[B1] Albina P. Durban N. Bertron A. Albrecht A. Robinet J. C. Erable B. (2021). Nitrate and nitrite bacterial reduction at alkaline pH and high nitrate concentrations, comparison of acetate versus dihydrogen as electron donors. J. Environ. Manage. 280, 111859. 10.1016/j.jenvman.2020.111859 33352382

[B2] Ceballos-Escalera A. Pous N. Chiluiza-Ramos P. Korth B. Harnisch F. Bañeras L.. (2021). Electro-bioremediation of nitrate and arsenite polluted groundwater. Water Res. 190, 116748. 10.1016/j.watres.2020.116748 33360100

[B3] Chaumeil P. A. Mussig A. J. Hugenholtz P. Parks D. H. (2020). GTDB-Tk: a toolkit to classify genomes with the genome taxonomy database. Bioinformatics 36, 1925–1927. 10.1093/bioinformatics/btz848 31730192 PMC7703759

[B4] Food and Agriculture Organization of the United Nations (2014). Small-Scale Aquaponic Food Production – Integrated Fish and Plant Farming. FAO Fisheries and Aquaculture Technical Paper. 978-92-5-108533-2.

[B5] Kanehisa M. Sato Y. Kawashima M. Furumichi M. Tanabe M. (2016). KEGG as a reference resource for gene and protein annotation. Nucleic Acids Res. 44, D457–D462. 10.1093/nar/gkv1070 26476454 PMC4702792

[B6] Kitzinger K. Koch H. Lücker S. Sedlacek C. J. Herbold C. Schwarz J.. (2018). Characterization of the first “Candidatus nitrotoga” isolate reveals metabolic versatility and separate evolution of widespread nitrite-oxidizing bacteria. MBio 9, e01186–e01118. 10.1128/mBio.01186-18 29991589 PMC6050957

[B7] Koch C. Korth B. Harnisch F. (2017). Microbial ecology-based engineering of microbial electrochemical technologies. Microb. Biotechnol. 11, 22–38. 10.1111/1751-7915.12802 28805354 PMC5743830

[B8] Korth B. Kretzschmar J. Bartz M. Kuchenbuch A. Harnisch F. (2020). Determining incremental coulombic efficiency and physiological parameters of early stage *Geobacter* spp. enrichment biofilms. PLoS ONE 15, e0234077. 10.1371/journal.pone.0234077 32559199 PMC7304624

[B9] Kracke F. Vassilev I. Krömer J. O. (2015). Microbial electron transport and energy conservation - the foundation for optimizing bioelectrochemical systems. Front. Microbiol. 6, e00575. 10.3389/fmicb.2015.00575 26124754 PMC4463002

[B10] Kretzschmar J. Harnisch F. (2021). Electrochemical impedance spectroscopy on biofilm electrodes – conclusive or euphonious? Curr. Opin. Electrochem. 29, 100757. 10.1016/j.coelec.2021.10075712194426

[B11] Li R. Li T. Wan Y. Zhang X. Liu X. Li R.. (2022). Efficient decolorization of azo dye wastewater with polyaniline/graphene modified anode in microbial electrochemical systems. J. Hazard. Mater. 421, 126740. 10.1016/j.jhazmat.2021.126740 34333409

[B12] Logan B. E. Rossi R. Ragab A. Saikaly P. E. (2019). Electroactive microorganisms in bioelectrochemical systems. Nat. Rev. Microbiol. 1, 307–319. 10.1038/s41579-019-0173-x 30846876

[B13] Mechichi T. Stackebrandt E. Fuchs G. (2003). Alicycliphilus denitrificans gen. nov., sp. nov., a cyclohexanol-degrading, nitrate-reducing β-proteobacterium. Int. J. Syst. Evol. Microbiol. 53, 147–152. 10.1099/ijs.0.02276-0 12661531

[B14] Morgado L. Paixão V. B. B. B. Schiffer M. Pokkuluri P. R. R. R. Bruix M. Salgueiro C. A. (2012). Revealing the structural origin of the redox-Bohr effect: the first solution structure of a cytochrome from Geobacter sulfurreducens. Biochem. J. 441, 179–187. 10.1042/BJ20111103 21861844

[B15] Newman J. S. Tobias C. W. (1962). Theoretical analysis of current distribution in porous electrodes. J. Electrochem. Soc. 109, 1183–1191. 10.1149/1.2425269

[B16] Pant D. Van Bogaert G. Diels L. Vanbroekhoven K. (2010). A review of the substrates used in microbial fuel cells (MFCs) for sustainable energy production. Bioresour. Technol. 101, 1533–1543. 10.1016/j.biortech.2009.10.017 19892549

[B17] Parks D. H. Rinke C. Chuvochina M. Chaumeil P. A. Woodcroft B. J. Evans P. N.. (2017). Recovery of nearly 8,000 metagenome-assembled genomes substantially expands the tree of life. Nat. Microbiol. 2, 1533–1542. 10.1038/s41564-017-0012-7 28894102

[B18] Peng L. Zhang X. T. Yin J. Xu S. Y. Zhang Y. Xie D. T.. (2016). Geobacter sulfurreducens adapts to low electrode potential for extracellular electron transfer. Electrochim. Acta 191, 743–749. 10.1016/j.electacta.2016.01.033

[B19] Pous N. Balaguer M. D. Colprim J. Puig S. (2018). Opportunities for groundwater microbial electro-remediation. Microb. Biotechnol. 11, 119–135. 10.1111/1751-7915.12866 28984425 PMC5743827

[B20] Pous N. Koch C. Colprim J. Puig S. Harnisch F. (2014). Extracellular electron transfer of biocathodes: revealing the potentials for nitrate and nitrite reduction of denitrifying microbiomes dominated by *Thiobacillus* sp. Electrochem. Commun. 49, 93–97. 10.1016/j.elecom.2014.10.011

[B21] Pous N. Koch C. Vilà-Rovira A. Balaguer M. D. Colprim J. Mühlenberg J.. (2015). Monitoring and engineering reactor microbiomes of denitrifying bioelectrochemical systems. RSC Adv. 5, 68326–68333. 10.1039/C5RA12113B

[B22] Pous N. Korth B. Osset-Álvarez M. Balaguer M. D. Harnisch F. Puig S. (2021). Electrifying biotrickling filters for the treatment of aquaponics wastewater. Bioresour. Technol. 319, 124221. 10.1016/j.biortech.2020.124221 33254451 PMC7547830

[B23] Prokhorova A. Kainuma M. Hiyane R. Boerner S. Goryanin I. (2021). Concurrent treatment of raw and aerated swine wastewater using an electrotrophic denitrification system. Bioresour. Technol. 322, 124508. 10.1016/j.biortech.2020.124508 33341711

[B24] Quejigo J. R. Korth B. Kuchenbuch A. Harnisch F. (2021). Redox potential heterogeneity in fixed-bed electrodes leads to microbial stratification and inhomogeneous performance. ChemSusChem 14, 1155–1165. 10.1002/cssc.202002611 33387375 PMC7986606

[B25] Quejigo J. R. Rosa L. F. M. Harnisch F. (2018). Electrochemical characterization of bed electrodes using voltammetry of single granules. Electrochem. Commun. 90, 78–82. 10.1016/j.elecom.2018.04.00933387375

[B26] Quejigo J. R. Sara Q. Sanz T. Esteve-Núñez A. Harnisch F. (2019). Bed electrodes in microbial electrochemistry: setup, operation and characterization. ChemTexts 5, 1–15. 10.1007/s40828-019-0078-3

[B27] Rabaey K. Clauwaert P. Aelterman P. Verstraete W. (2005). Tubular microbial fuel cells for efficient electricity generation. Environ. Sci. Technol. 39, 8077–8082. 10.1021/es050986i 16295878

[B28] Rodríguez Arredondo M. Kuntke P. Jeremiasse A. W. Sleutels T. H. J. A. Buisman C. J. N. Ter Heijne A. (2015). Bioelectrochemical systems for nitrogen removal and recovery from wastewater. Environ. Sci. Water Res. Technol. 1, 22–33. 10.1039/C4EW00066H

[B29] Ryan M. P. Pembroke J. T. Adley C. C. (2007). Ralstonia pickettii in environmental biotechnology: potential and applications. J. Appl. Microbiol. 103, 754–764. 10.1111/j.1365-2672.2007.03361.x 17897177

[B30] Safonov A. V. Babich T. L. Sokolova D. S. Grouzdev D. S. Tourova T. P. Poltaraus A. B.. (2018). Microbial community and *in situ* bioremediation of groundwater by nitrate removal in the zone of a radioactive waste surface repository. Front. Microbiol. 9, e01985. 10.3389/fmicb.2018.01985 30190715 PMC6115527

[B31] Schröder U. Harnisch F. Angenent L. T. (2015). Microbial electrochemistry and technology: terminology and classification. Energy Environ. Sci. 8, 513–519. 10.1039/C4EE03359K

[B32] Seemann T. (2014). Prokka: Rapid prokaryotic genome annotation. Bioinformatics 30, 2068–2069. 10.1093/bioinformatics/btu153 24642063

[B33] Seitzinger S. P. Phillips L. (2017). Nitrogen stewardship in the Anthropocene. Science. 357, 350–351. 10.1126/science.aao0812 28751593

[B34] Sevda S. Sreekishnan T. R. Pous N. Puig S. Pant D. (2018). Bioelectroremediation of perchlorate and nitrate contaminated water: a review. Bioresour. Technol. 255, 331–339. 10.1016/j.biortech.2018.02.005 29439851

[B35] Shaw D. R. Ali M. Katuri K. P. Gralnick J. A. Reimann J. Mesman R.. (2020). Extracellular electron transfer-dependent anaerobic oxidation of ammonium by anammox bacteria. Nat. Commun. 11, 1–12. 10.1038/s41467-020-16016-y 32345973 PMC7188810

[B36] Srivastava P. Yadav A. K. Garaniya V. Lewis T. Abbassi R. Khan S. J. (2020). Electrode dependent anaerobic ammonium oxidation in microbial fuel cell integrated hybrid constructed wetlands: a new process. Sci. Total Environ. 698, 134248. 10.1016/j.scitotenv.2019.134248 31494423

[B37] Tiedje J. M. (1988). Ecology of denitrification and dissimilatory nitrate reduction to ammonium, in Environmental Microbiology of Anaerobes, ed. Zehnder A. J. B. (New York, NY: John Wiley and Sons), 179–244.

[B38] Uritskiy G. V. DiRuggiero J. Taylor J. (2018). MetaWRAP—a flexible pipeline for genome-resolved metagenomic data analysis. Microbiome 6, 1–13. 10.1186/s40168-018-0541-1 30219103 PMC6138922

[B39] Vilajeliu-Pons A. Koch C. Balaguer M. D. Colprim J. Harnisch F. Puig S. (2018). Microbial electricity driven anoxic ammonium removal. Water Res. 130, 168–175. 10.1016/j.watres.2017.11.059 29220717

[B40] Vilar-Sanz A. Pous N. Puig S. Balaguer M. D. Colprim J. Bañeras L. (2018). Denitrifying nirK-containing alphaproteobacteria exhibit different electrode driven nitrite reduction capacities. Bioelectrochemistry 121, 74–83. 10.1016/j.bioelechem.2018.01.007 29413866

[B41] Virdis B. Rabaey K. Yuan Z. Keller J. (2008). Microbial fuel cells for simultaneous carbon and nitrogen removal. Water Res. 42, 3013–3024. 10.1016/j.watres.2008.03.017 18466949

[B42] Wang K. Zhang S. (2019). Extracellular electron transfer modes and rate-limiting steps in denitrifying biocathodes. Environ. Sci. Pollut. Res. 26, 16378–16387. 10.1007/s11356-019-05117-x 30982192

[B43] Wang X. Aulenta F. Puig S. Esteve-Núñez A. He Y. Mu Y.. (2020). Microbial electrochemistry for bioremediation. Environ. Sci. Ecotechnology 1, 100013. 10.1016/j.ese.2020.100013 PMC9488016 36160374

[B44] Yadav A. K. Dash P. Mohanty A. Abbassi R. Mishra B. K. (2012). Performance assessment of innovative constructed wetland-microbial fuel cell for electricity production and dye removal. Ecol. Eng. 47, 126–131. 10.1016/j.ecoleng.2012.06.029

[B45] Yin H. Yang C. Jia Y. Chen H. Gu X. (2018). Dual removal of phosphate and ammonium from high concentrations of aquaculture wastewaters using an efficient two-stage infiltration system. Sci. Total Environ. 635, 936–946. 10.1016/j.scitotenv.2018.04.218 29710615

[B46] Yu L. Yuan Y. Chen S. Zhuang L. Zhou S. (2015). Direct uptake of electrode electrons for autotrophic denitrification by *Thiobacillus denitrificans*. Electrochem. Commun. 60, 126–130. 10.1016/j.elecom.2015.08.025

[B47] Zhang H. Zhao Z. Chen S. Kang P. Wang Y. Feng J.. (2018). Paracoccus versutus KS293 adaptation to aerobic and anaerobic denitrification: insights from nitrogen removal, functional gene abundance, and proteomic profiling analysis. Bioresour. Technol. 260, 321–328. 10.1016/j.biortech.2018.03.123 29631182

[B48] Zhao J. J. Zhang J. Sun L. Zhang R. J. Zhang C. W. Yin H. Q.. (2017). *Rhizobium oryziradicis* sp. Nov., isolated from rice roots. Int. J. Syst. Evol. Microbiol. 67, 963–968. 10.1099/ijsem.0.001724 27959784

