# Supplementary material for: Electrochemical and Microbial Dissection of Electrified Biotrickling Filters
Source: Front Microbiol. 2022 May 31;13:869474. doi: 10.3389/fmicb.2022.869474 (PMC9197458; doi:10.3389/fmicb.2022.869474)
Supplement: Supplementary file 1 [file Data_Sheet_1.zip › Supplementary Material 1.pdf]

Supplementary Material

Electrochemical and microbial dissection of electrified biotrickling filters

Benjamin Korth[1,*], Narcís Pous[2], Richard Hönig[1], Philip Haus[1], Felipe Borim Corrêa[1], Ulisses Nunes da Rocha[1], Sebastià Puig[2], Falk Harnisch[1,*]

[1]Department of Environmental Microbiology, Helmholtz Centre for Environmental Research, Leipzig, Germany,

[2]Laboratory of Chemical and Environmental Engineering (LEQUiA), Institute of the Environment, University of Girona, Girona, Spain

*** Correspondence:**
Benjamin Korth
benjamin.korth@ufz.de

Falk Harnisch
falk.harnisch@ufz.de

1 Workflow for sampling granules from electrified biotrickling filters

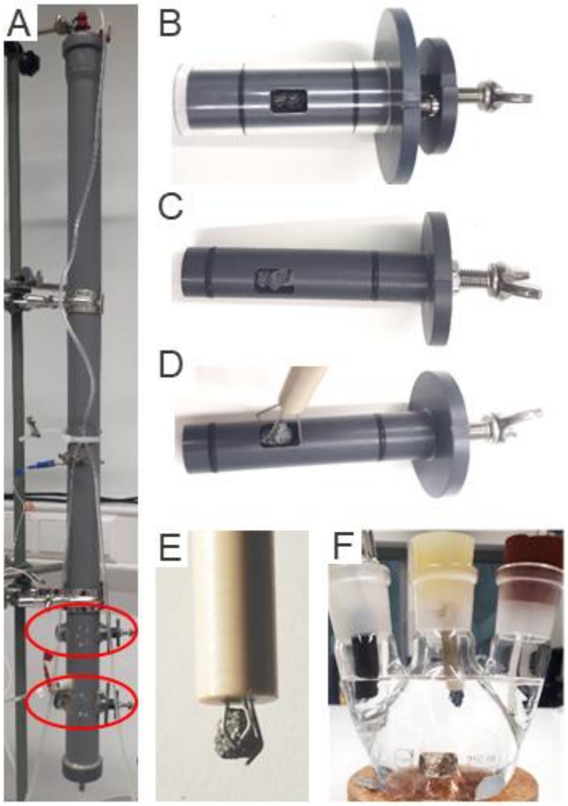

Figure S1 A) Electrified biotrickling filters with integrated sampling units (red circles). B) Sampling unit consisting of three cylinders with sampled granule. C) Inner cylinder with sampled granule. D-E) Grabbing of sampled granule with e-Clamp. E) Four-neck round-bottom flask with inserted e-Clamp, reference electrode, and counter electrode chamber.

2 Process parameters of all reactors

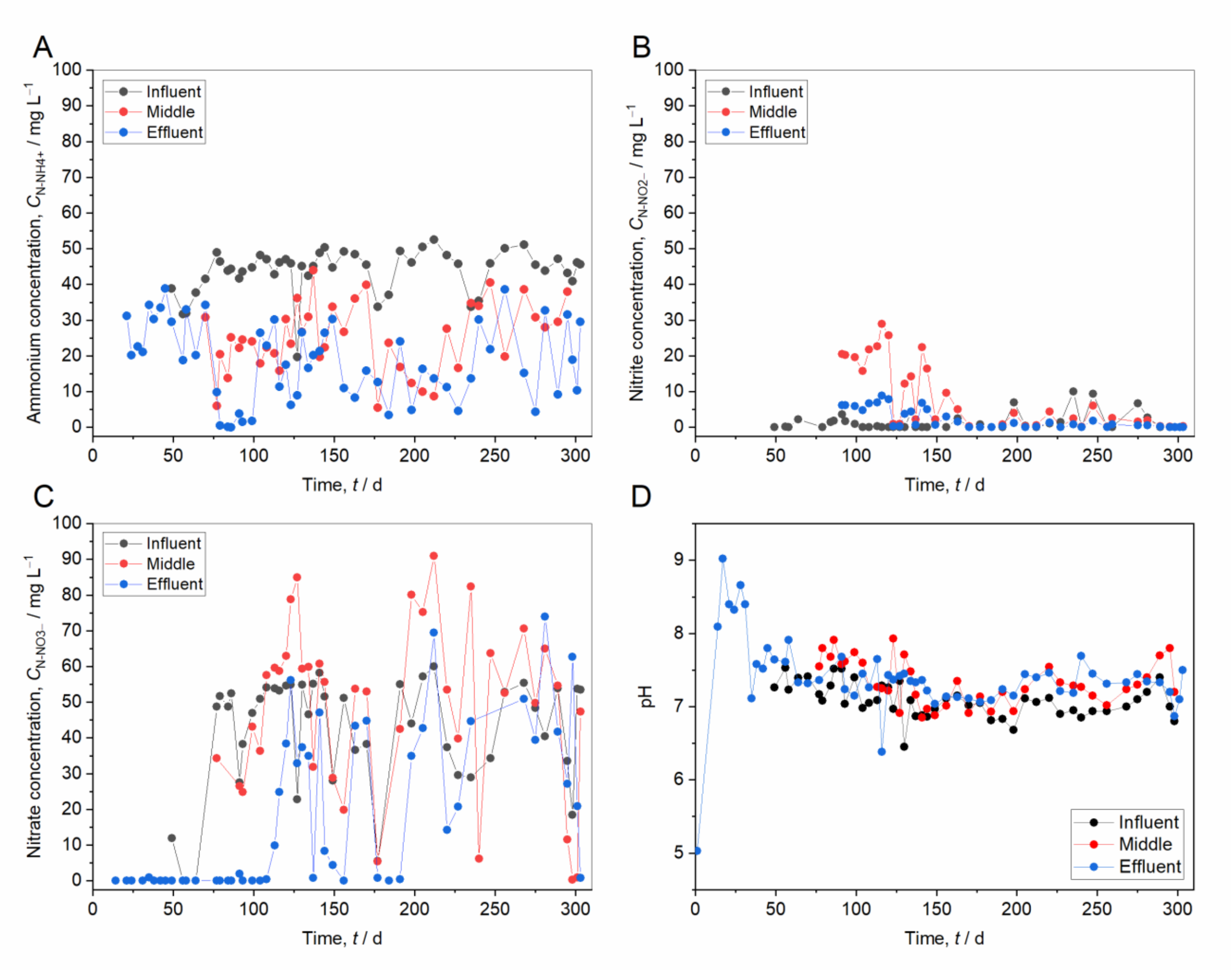

Figure S2 Overview of process parameters of electrified biotrickling filter with integrated sampling units (Reactor 1) during inoculation and adaptation (day 1-100) and operational phase (day 101-300). A) Ammonium concentration, B) nitrite concentration, C) nitrate concentration, D) pH.

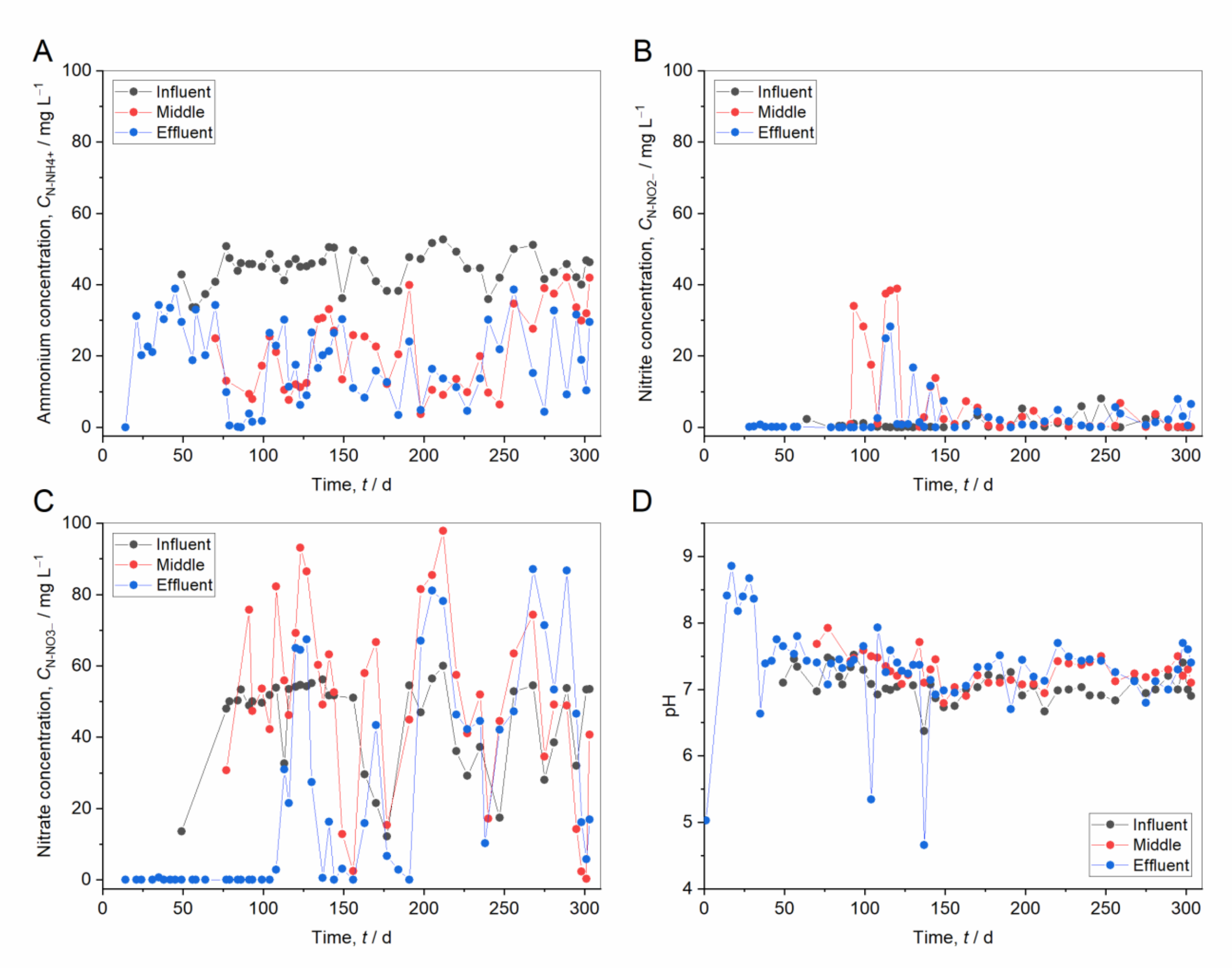

Figure S3 Overview of process parameters of electrified biotrickling filter with integrated sampling units (Reactor 2) during inoculation and adaptation (day 1-100) and operational phase (day 101-300). A) Ammonium concentration, B) nitrite concentration, C) nitrate concentration, D) pH.

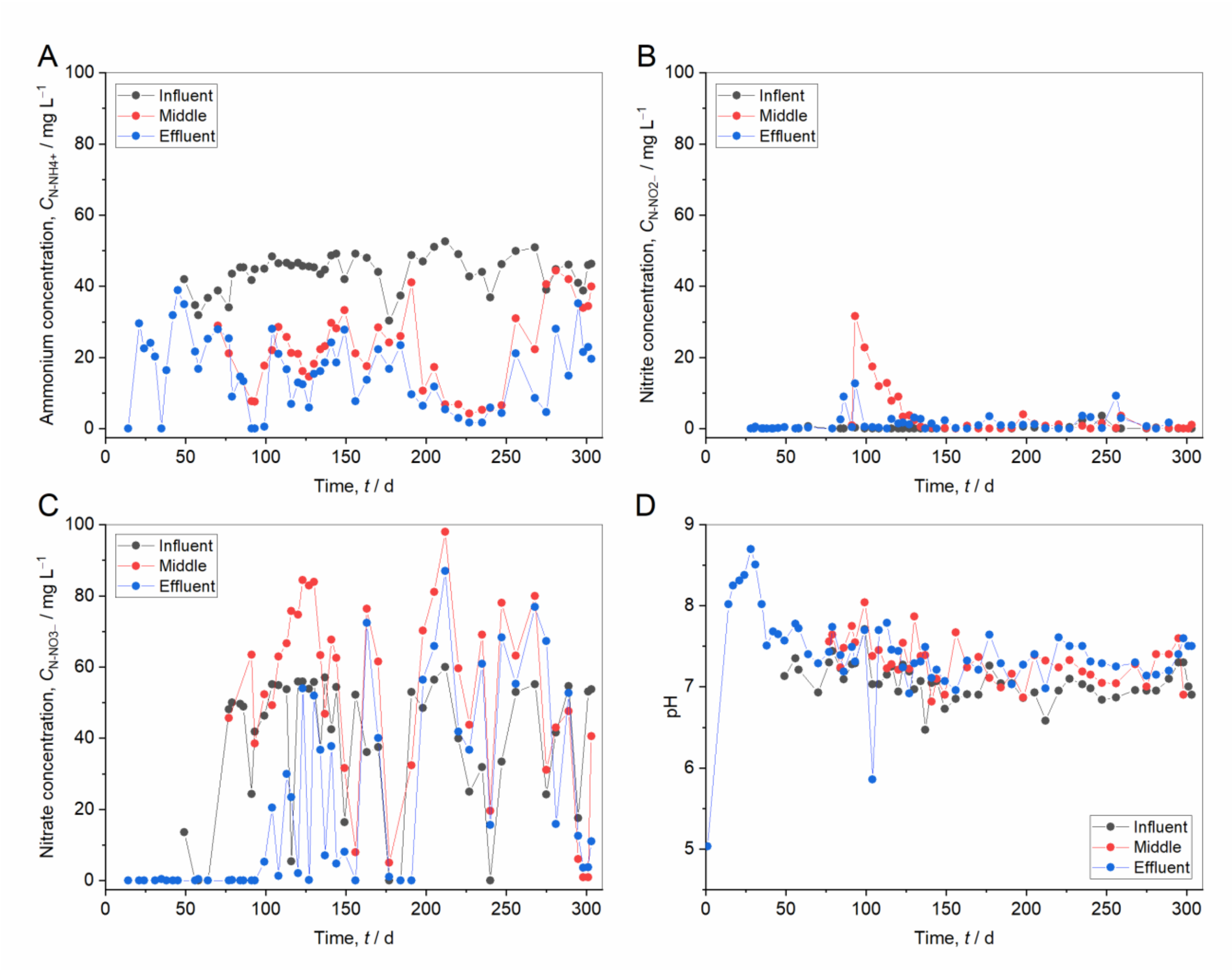

Figure S4 Overview of process parameters of electrified biotrickling filter with integrated sampling units (Reactor 3) during inoculation and adaptation (day 1-100) and operational phase (day 101-300). A) Ammonium concentration, B) nitrite concentration, C) nitrate concentration, D) pH.

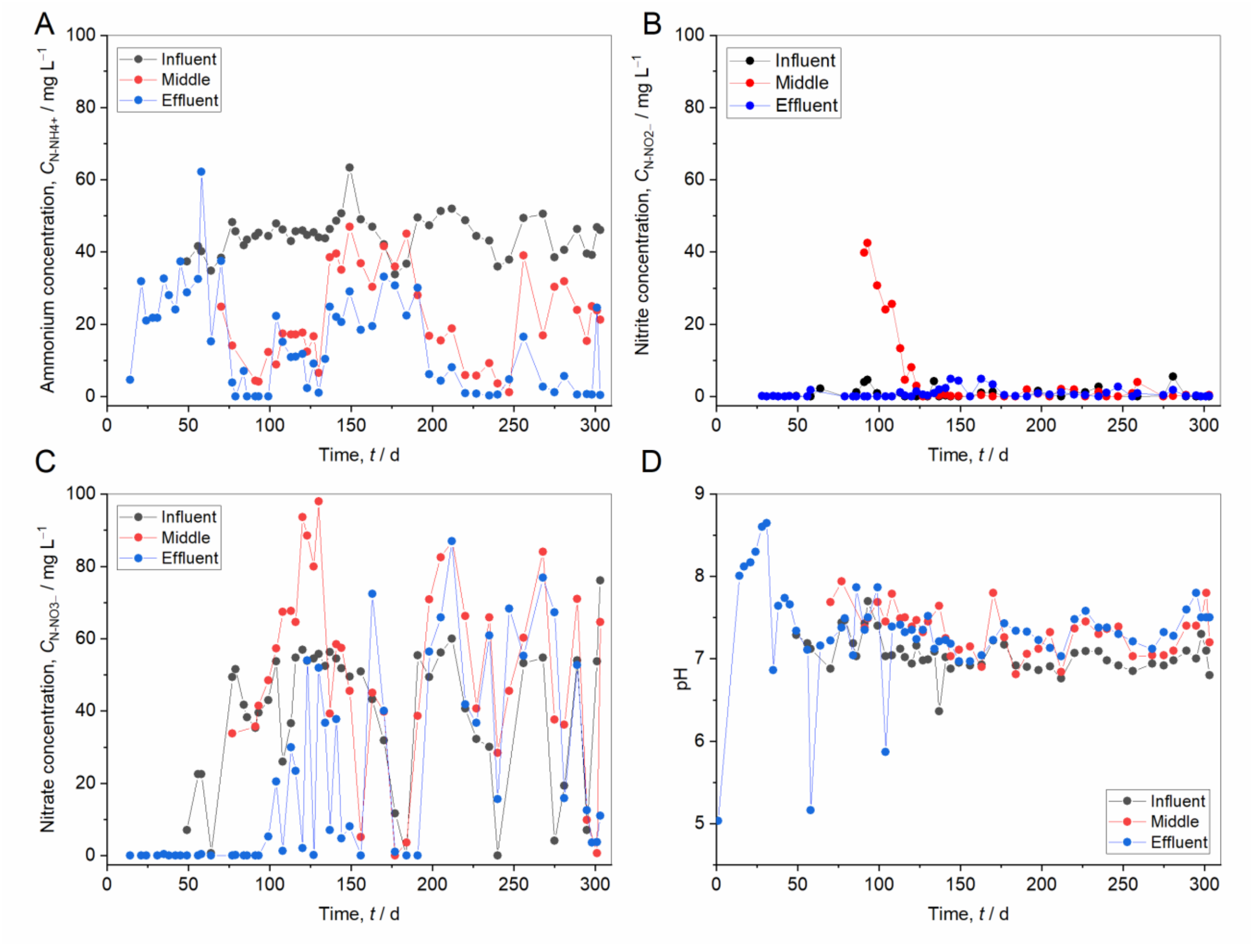

Figure S5 Overview of process parameters of electrified biotrickling filter (control reactor) during inoculation and adaptation (day 1-100) and operational phase (day 101-300). A) Ammonium concentration, B) nitrite concentration, C) nitrate concentration, D) pH.

3 Cyclic voltammetry with granules sampled from the vertical axes of electrified biotrickling filters at the end of the operational phase

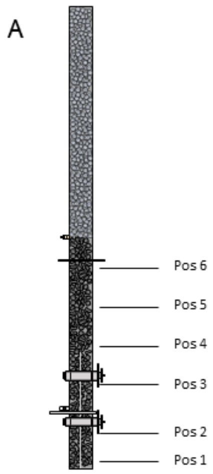

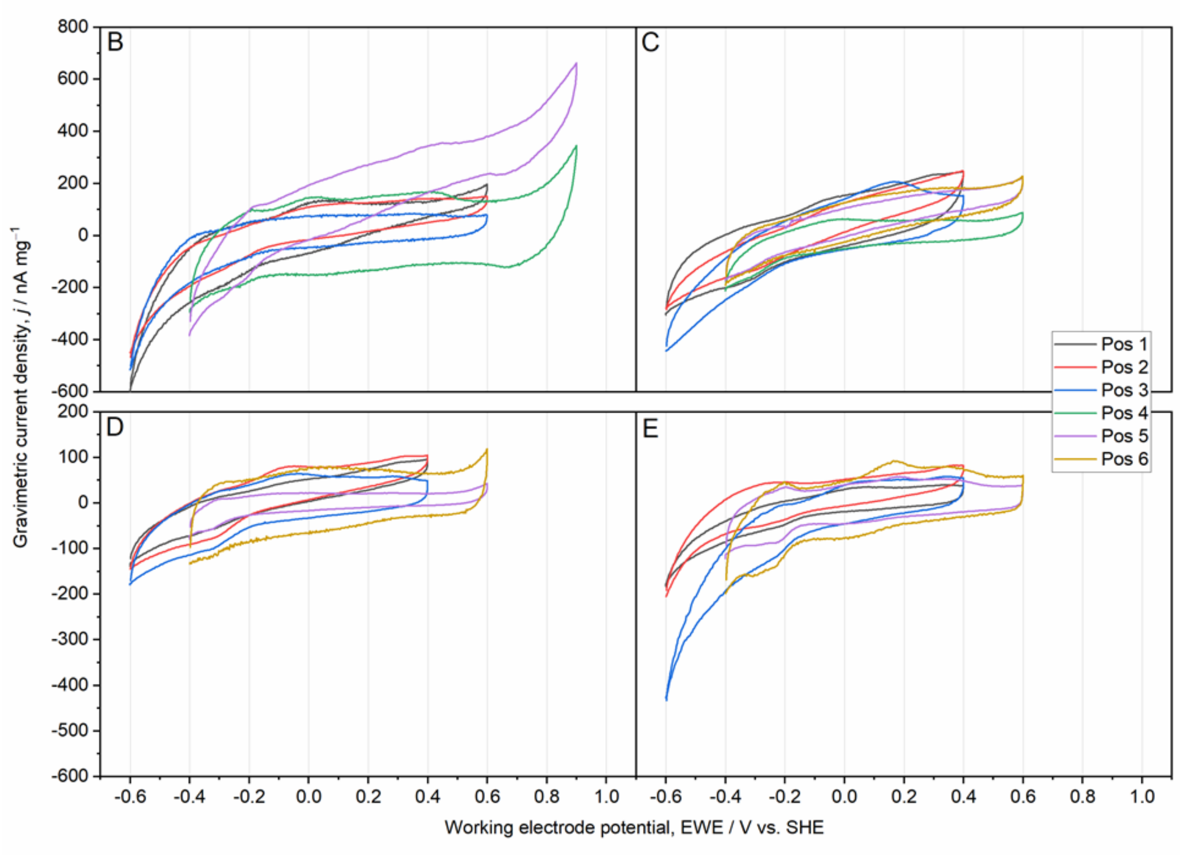

Figure S6 E-Clamp analysis of granules sampled from the vertical axis of all reactors at the end of the operational phase. A) Sample scheme: Granules were sampled from six positions equally distributed along the vertical axis. B-D) Electrified biotrickling filter, reactor 1-3. E) Control reactor. Granules extracted from Pos 6 (Reactor 1) and Pos 4 (Reactor 3 and control reactor) exhibited no evaluable electrochemical signal.

4 Cyclic voltammetry - Control experiments and exemplary anodic redox peaks

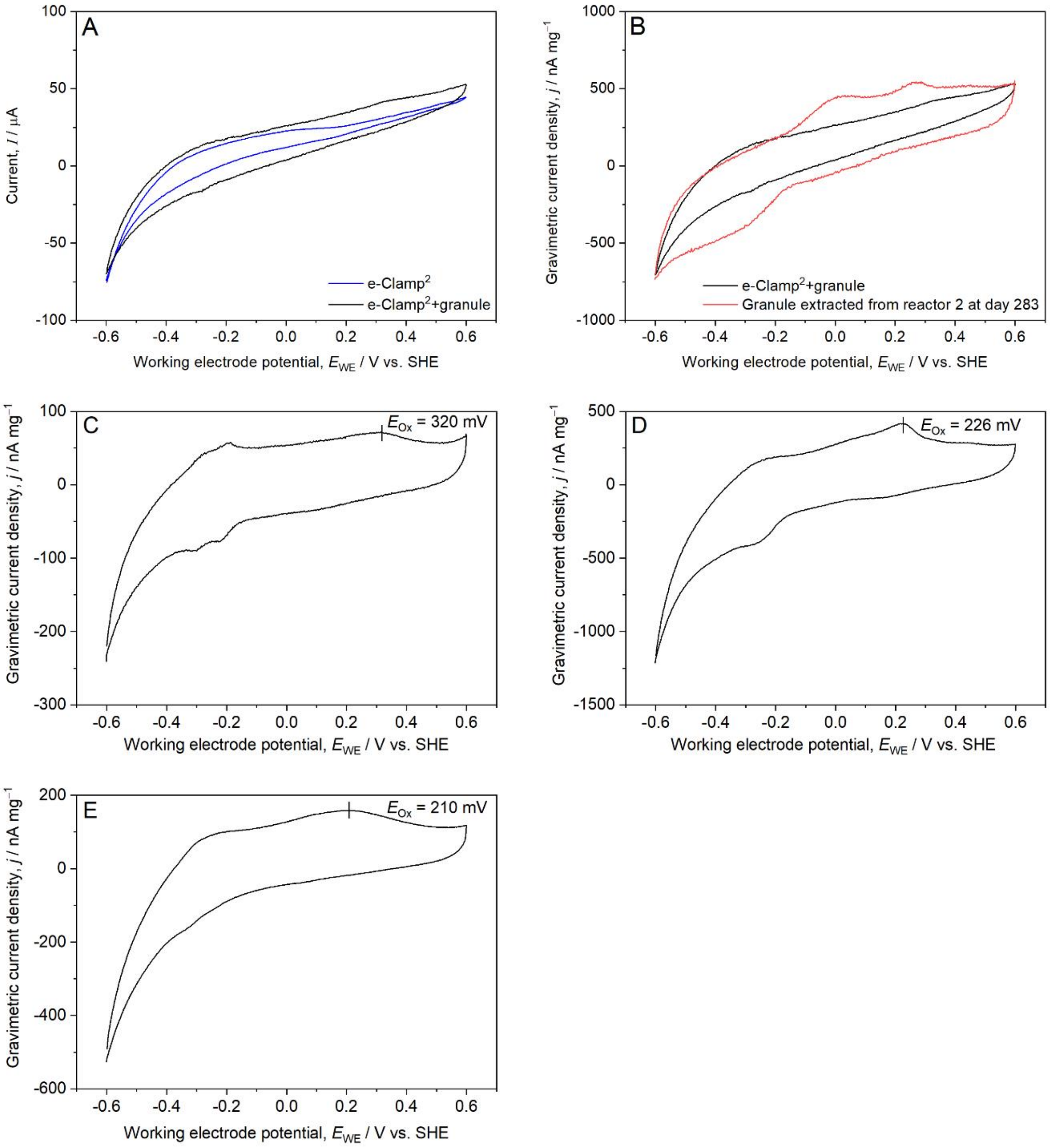

Figure S7 Cyclic voltammograms of A) blank e-Clamp without granule and e-Clamp with a non-inoculated granule. CV was performed at pH 6.8. B) e-Clamp with a non-inoculated granule and with a sampled granule from reactor 2, CV was performed at pH 7. C-E) Exemplary cyclic voltammograms of granules that were sampled from the modified biotrickling filters during the operational phase. The CV show anodic peaks that were occasionally observed. A) Reactor 1, sampling unit 1 (close to current collector), pH 7.2 day 253. B) Reactor 2, sampling unit 1 (close to current collector), pH 6.9 day 238. C) Reactor 3, sampling unit 2, pH 7.2 day 247. The identified oxidation potentials (E_{Ox}) are indicated. Scan rate was 1 mV s^{-1}, 3rd scans are shown.

5 Analysis of the pH influence on the gravimetric current density with correction for the formal potential.

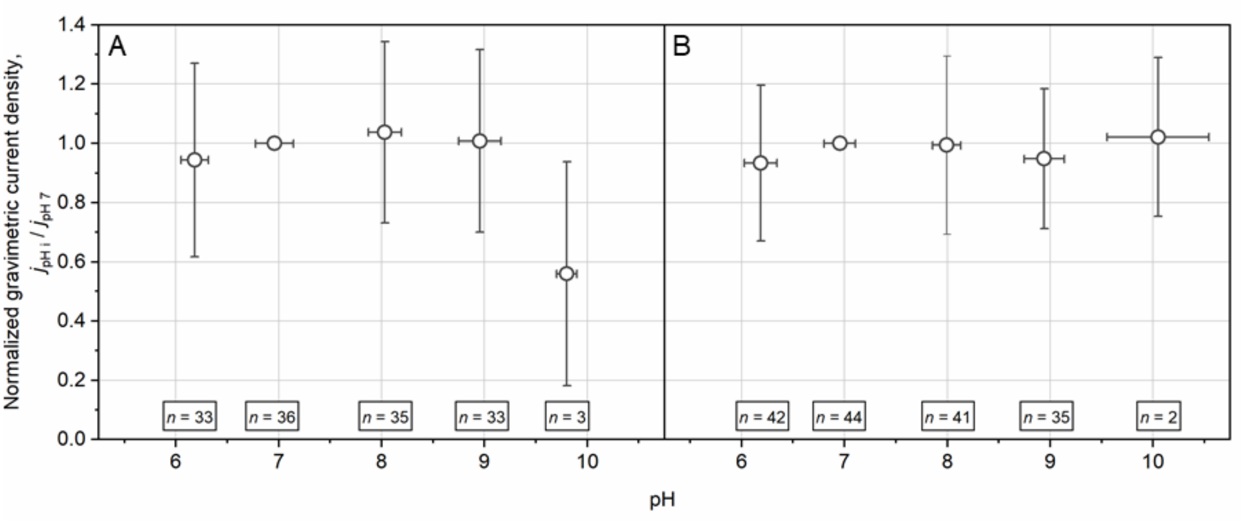

Figure S8 Normalized gravimetric current density (j) of redox sites A) E_{f1} and B) E_{f2} as a function of pH. j was quantified at the formal potentials E_{f1} and E_{f2} that were individually identified for every pH ($j_{pH\,i}$) and normalized to the gravimetric current density at pH 7 ($j_{pH\,7}$). Per definition, the normalized gravimetric current density is 1 at pH 7. Errors bars indicate standard deviations. n indicates the number of analyzed granules.

6 TRFLP analysis with granules sampled from reactors 1 and 2 during the operational phase

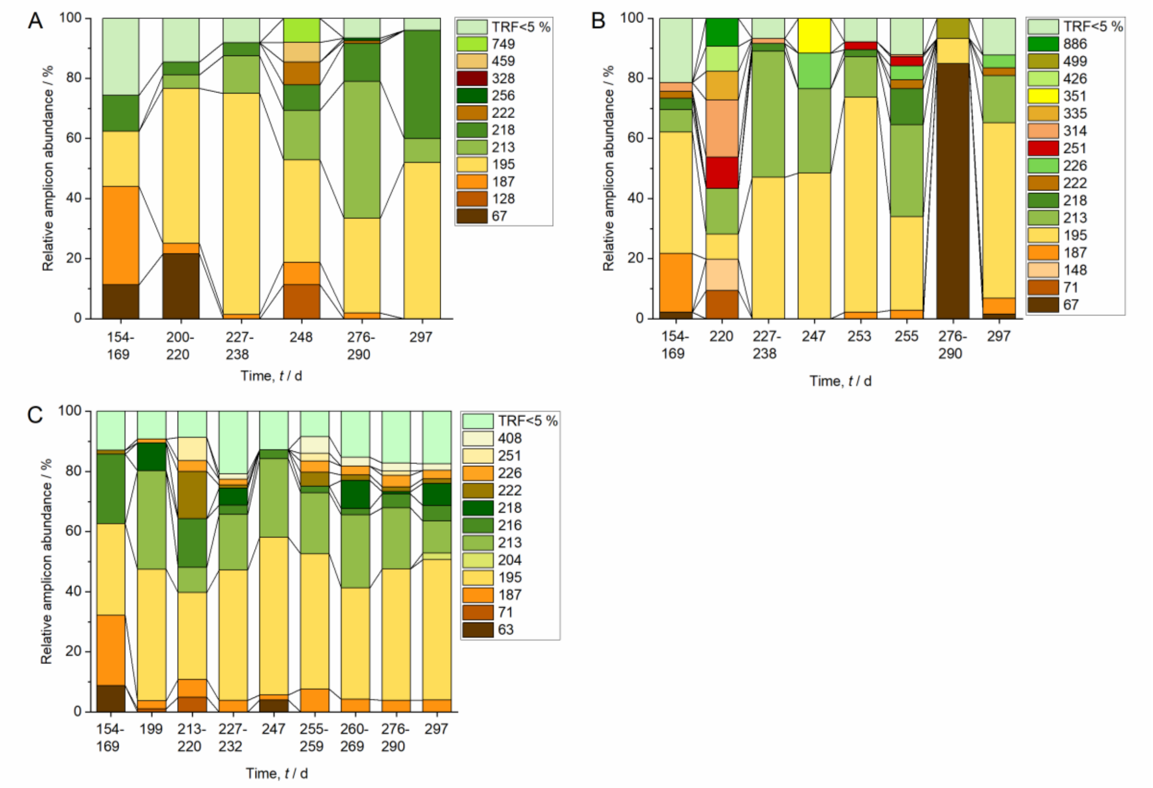

Figure S9 Analysis of the microbial community attached to the graphite granules extracted from both sampling units of two electrified biotrickling filters during the operational phase: A) reactor 1, B) reactor 2, and C) reactor 3. A partial sequence of 16S rRNA genes of bacteria was analyzed via TRFLP for revealing the relative amplicon abundances. Few terminal restriction fragments (TRF, 195 base pairs (bp), 213 bp, and 218 bp) were found to dominate the microbial community of the granule bed during the whole operational phase indicating the existence of a core community. TRF that exhibited a relative abundance of lower than 5 % during the whole operational phase were joined together. Restriction enzyme *Hae*III was used for analysis.

7 TRFLP analysis with granules sampled from the vertical axes of all reactors at the end of the operational phase

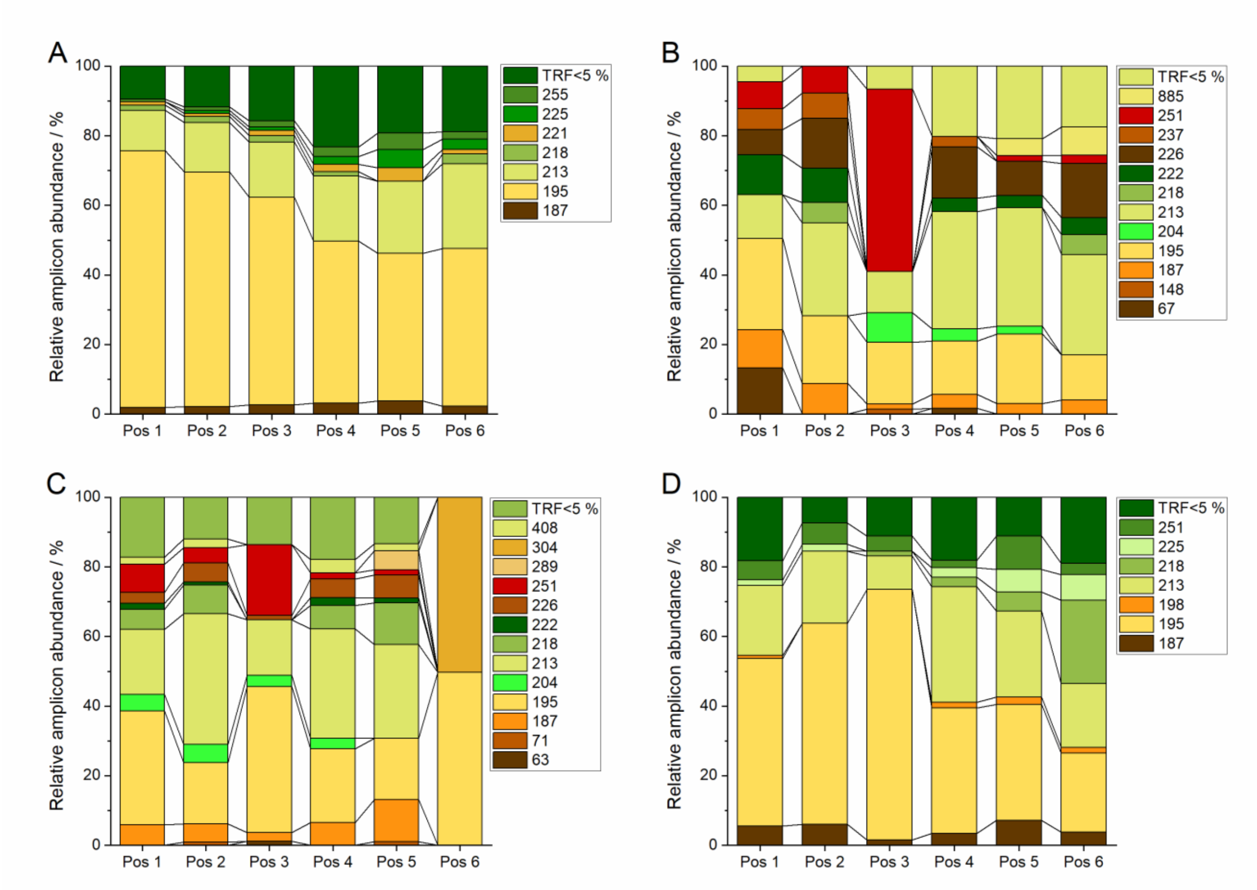

Figure S10 Analysis of the microbial community attached to the graphite granules extracted from the vertical axes of all reactor sat the end of the operational phase. A) Reactor 1, B) reactor 2, C) reactor 3, D) control reactor. A partial sequence of 16S rRNA genes of bacteria was analyzed via TRFLP for revealing the relative amplicon abundances. Interestingly, similar TRF patterns appear in all positions of the electrified biotrickling filters during the vertical analysis at the end of the operational phase (TRF 195 bp, 213 bp, and 218 bp dominated the microbial community). This indicates that the incorporated CC (metal mesh covering ca. 50 % of the vertical axis of the granule bed) led to a homogeneous potential distribution resulting in a comparable distribution of the microbial community. However, in addition to the redox potential gradient, other gradients (e.g., ammonia, nitrate, intermediates of nitrogen pathways, oxygen) exist within the granule bed that influence microbial growth and potentially covered a possible microbial stratification induced by the redox potential. TRF that exhibited a relative abundance of lower than 5 % during the whole operational phase were joined together. Restriction enzyme *Hae*III was used. Sample scheme is illustrated in Figure S1A.
